# Potent Host-Directed Small-Molecule Inhibitors of Myxovirus RNA-Dependent RNA-Polymerases

**DOI:** 10.1371/journal.pone.0020069

**Published:** 2011-05-16

**Authors:** Stefanie A. Krumm, J. Maina Ndungu, Jeong-Joong Yoon, Melanie Dochow, Aiming Sun, Michael Natchus, James P. Snyder, Richard K. Plemper

**Affiliations:** 1 Department of Pediatrics, Emory University School of Medicine, Atlanta, Georgia, United States of America; 2 Children's Healthcare of Atlanta, Atlanta, Georgia, United States of America; 3 Emory Institute for Drug Discovery, Emory University, Atlanta, Georgia, United States of America; 4 Department of Chemistry, Emory University, Atlanta, Georgia, United States of America; 5 Department of Microbiology & Immunology, Emory University School of Medicine, Atlanta, Georgia, United States of America; College of Medicine, Hallym University, Republic of Korea

## Abstract

Therapeutic targeting of host cell factors required for virus replication rather than of pathogen components opens new perspectives to counteract virus infections. Anticipated advantages of this approach include a heightened barrier against the development of viral resistance and a broadened pathogen target spectrum. Myxoviruses are predominantly associated with acute disease and thus are particularly attractive for this approach since treatment time can be kept limited. To identify inhibitor candidates, we have analyzed hit compounds that emerged from a large-scale high-throughput screen for their ability to block replication of members of both the orthomyxovirus and paramyxovirus families. This has returned a compound class with broad anti-viral activity including potent inhibition of different influenza virus and paramyxovirus strains. After hit-to-lead chemistry, inhibitory concentrations are in the nanomolar range in the context of immortalized cell lines and human PBMCs. The compound shows high metabolic stability when exposed to human S-9 hepatocyte subcellular fractions. Antiviral activity is host-cell species specific and most pronounced in cells of higher mammalian origin, supporting a host-cell target. While the compound induces a temporary cell cycle arrest, host mRNA and protein biosynthesis are largely unaffected and treated cells maintain full metabolic activity. Viral replication is blocked at a post-entry step and resembles the inhibition profile of a known inhibitor of viral RNA-dependent RNA-polymerase (RdRp) activity. Direct assessment of RdRp activity in the presence of the reagent reveals strong inhibition both in the context of viral infection and in reporter-based minireplicon assays. *In toto*, we have identified a compound class with broad viral target range that blocks host factors required for viral RdRp activity. Viral adaptation attempts did not induce resistance after prolonged exposure, in contrast to rapid adaptation to a pathogen-directed inhibitor of RdRp activity.

## Introduction

Myxoviruses are enveloped, negative-strand RNA viruses that are transmitted through the respiratory route. The orthomyxovirus family comprises five different genera of which the influenza viruses are clinically most relevant. Of the paramyxoviridae, respiratory syncytial virus (RSV), measles virus (MeV), mumps virus (MuV), human parainfluenzaviruses (HPIV) and the recently emerged, highly pathogenic zoonotic henipaviruses constitute major human pathogens [1]. Although clinical complications associated with some myxoviruses involve persistent infections, the viruses predominantly induce acute respiratory or systemic disease.

Collectively, myxoviruses are responsible for the majority of human morbidity and mortality due to viral respiratory illness globally [2], [3]. In particular, influenza virus is the leading cause of morbidity and mortality from respiratory disease in North America despite the existence of vaccine prophylaxis. This is due to the fact that the vaccines currently in use reduce illness in approximately 70% of healthy adults when homologous to the prevalent circulating virus, but protection in the elderly reaches only approximately 40%. Vaccine efficacy is reduced substantially when the circulating strains differ from those constituting the vaccine [2].

Despite extensive research and in contrast to, for instance, MeV and MuV, no vaccines are currently available against several major pathogens of the paramyxovirus family such as RSV or different HPIVs. Infection with RSV is the leading cause of pneumonia and bronchiolitis in infants, both associated with significant mortality, while HPIV types 1 and 2 are the primary cause of croup syndrome and can likewise result in serious lower respiratory diseases such as pneumonia and bronchiolitis [4], [5].

The availability of effective antiviral therapy for most clinically significant myxovirus infections is limited. Licensed neuraminidase inhibitors for influenza therapy, Zanamivir and Oseltamivir, show efficacy when administered within a 48-hour window after the onset of symptoms, but are increasingly compromised by pre-existing or emerging viral resistance [6], [7], [8]. Ribavirin, although approved for RSV treatment, shows limited utility due to efficacy and toxicity issues [9]. The polyclonal immunoglobulin RSV-IVIG [10] and the humanized monoclonal antibody Synagis [11] provide RSV prophylaxis, but use is limited to high-risk pediatric patients. Considering the high mutation rates seen in particular with RNA viruses [12], [13], the development of novel types of myxovirus inhibitors that circumvent the rapid development of resistance is highly desirable.

Of the strategies conceivable towards this goal, targeting host factors required for completion of the viral life cycle rather than pathogen-encoded factors directly has received heightened interest in recent years [14], [15]. This approach is expected to establish a significant barrier against spontaneous viral escape from inhibition, since individual viral mutations are less likely to compensate for the loss of an essential host cofactor than to prevent high-affinity binding of a conventional, pathogen-directed antiviral. Given some degree of overlap of host cell pathways required for successful replication of related viral pathogens, host-directed antiviral approaches also have the potential to move beyond the one-bug one-drug paradigm by broadening the pathogen target range of a chemical scaffold.

Naturally, targeting host factors for antiviral therapy bears an inherently higher potential for undesirable drug-induced side effects than conventional pathogen-directed strategies. While the approach is nevertheless under investigation for the treatment of chronic viral infections such as HSV-1 and HIV-1 [16], [17], an application to the inhibition of infections by pathogens predominantly associated with severe acute disease, such as most members of the myxovirus families, is anticipated to render drug-related side effects tolerable to some extent, since the necessary treatment time and concomitant host exposure to the drug remain limited. In the case of influenza infections, for instance, typical neuraminidase inhibitor regimens consist of twice daily administration for a five-day period for treatment, or a 10-day period for prophylaxis [18].

Relying on a broadened anti-myxovirus target spectrum as the main selection criterion in secondary screening assays, we have mined results of a recently completed high throughput chemical library screen [19] to identify hit candidates with a possible host-directed mechanism of action. This has yielded a compound class with broad anti-viral activity, which was subjected to synthetic scaffold optimization, quantification of active concentrations for a select group of clinically relevant ortho- and paramyxovirus family members, testing against a panel of exposed host cells of different species origin, and characterization of the compound-induced point-of-arrest in viral life cycle progression. Viral adaptation to growth in the presence of inhibitor has been employed to compare escape rates from inhibition by this new compound class with those from a well-characterized, pathogen-directed antiviral.

## Results

To identify small-molecule hit candidates that block the myxovirus life cycle through a host-directed mechanism, we analyzed the results of a high-throughput cell-based anti-MeV screen of a 137,500-entry library of the NIH diversity set that we recently reported [19]. The primary screening agent, serving as the myxovirus representative, was the wild type MeV isolate MVi/Alaska.USA/16.00 (MeV-Alaska). It was chosen based on its ease of growth and readily quantifiable cytopathic effect in the automated system [19], [20]. In search of candidates with a host-directed antiviral profile, we anticipated three distinct features of desirable compounds: a) potent inhibition of virus replication at the screening concentration (3.3 µM); b) a primary screening score, representative of the selectivity index (CC_50_/EC_50_), close to the cut-off value for hit candidates due to some anticipated host-cell interference ( = 1.9); and c) a broadened viral target spectrum in counterscreening assays that extends to other pathogens of the myxovirus families.

### Identification of a chemical scaffold with broad anti-viral activity

**Figure 1:**
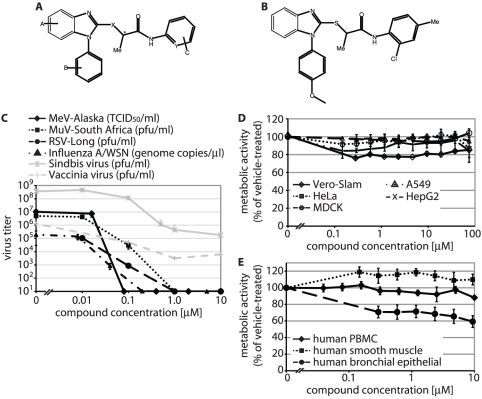
Identification of a chemical scaffold with broad anti-myxovirus activity. Chemical structures of the identified scaffold (**A**) and the current lead analog JMN3-003 (**B**). **C**) Dose-response curves for JMN3-003 and MeV-Alaska, MuV-South Africa, RSV Long, influenza A/WSN (H1N1), sindbis virus and vaccinia virus. Titers of cell-associated progeny viruses were determined by TCID_50_ titration (MeV) or plaque assay (MuV, RSV, sindbis virus, vaccinia virus). For influenza virus, genome copy numbers of released progeny particles were quantified through TaqMan RT-PCR. Titers of released sindbis virus particles were determined by plaque assay. Values reflect averages of at least three experiments ± SD, vaccinia virus titers were determined in duplicate. **D and E**) Assessment of metabolic activity of cells after incubation of different established cell lines (D) or primary human cells (E) in the presence of JMN3-003 for 24 hours. Results for human (HeLa, A549, HepG2), primate (Vero-Slam), and canine (MDCK) cell lines and primary human cells (PBMC, smooth muscle, bronchial epithelial) are shown. Values reflect averages of four replicates ± SD.

When inhibition of paramyxovirus family members was assessed, six compounds efficiently blocked the closely related canine distemper virus (CDV) and the more distantly related human parainfluenzavirus type 3 (HPIV3) in addition to MeV-Alaska, while leaving cell metabolic activity essentially unaffected [19]. Of these independent hits, three share a common molecular scaffold ([19] and figure 1A). Since HTS scores of these analogs best matched the target criteria and antiviral activity was highest in this group [19], we subjected them to further characterization and developmental efforts. Synthetic optimization and structural confirmation of the scaffold returned a lead analog JMN3-003 (figures 1B and S1), which showed potent activity against MeV, a selection of clinically significant members of the para- and orthomyxovirus families, and, albeit to a lesser degree, representatives of positive strand RNA virus (sindbis virus of the *Alphaviridae*) and DNA virus (vaccinia virus of the *Poxviridae*) families (figure 1C, inhibitory concentrations for a larger panel of myxovirus family members are summarized in table 1). As observed for the primary hit compound, metabolic activity of different established cell lines exposed to JMN3-003 was unchanged at 75 µM, the highest assessable concentration based on solubility of the substance in growth media (figure 1D and table 1). Of different primary human cells examined, metabolic activity was unaffected (PBMCs, smooth muscle cells) or only slightly affected (bronchial epithelial cells) by the compound (figure 1E). These data support potent anti-myxovirus activity of the compound with active concentrations ranging from 10 to 80 nM depending on the target virus.

**Table 1:** Active (EC_50_) and toxic (CC_50_, determined on Vero-Slam cells) concentrations of JMN3-003 against a selection of clinically relevant para- and orthomyxovirus family members in comparison with active concentrations of AS-136A, a previously characterized, MeV-specific inhibitor of the viral RdRp complex [20], [36].

Compound	Orthomyxoviridae^a^				Paramyxoviridae				Toxicity
	Influenza A/WSN	Influenza A/PR/8/34	SOI Influenza A/Texas	SOI Influenza A/Mexico	RSV^b^ (Long)	MuV^b^ (S. Africa)	HPIV3^b^	MeV^c^ (Alaska)	Metabolic activity
	**EC_50_ [µM]**								**CC_50_ [µM]** d
JMN3-003	0.01±0.008	0.01±0.001	0.04±0.01	0.01±0.003	0.07±0.01	0.033±0.031	0.08±0.01	0.03±0.02	>75
AS-136A	none detected	ND	ND	ND	none detected	none detected	none detected	0.03^e^ (0.01–0.05)	>75

a For influenza virus titration, genome copy numbers of released progeny particles were quantified by TaqMan RT-PCR.

b Titered through plaque assaying.

c Titered by TCID_50_ titration.

d Highest concentration assessed 75 µM.

e 95% confidence interval.

ND: not determined.

### Antiviral activity of lead compound JMN3-003 is host cell-specific

**Figure 2:**
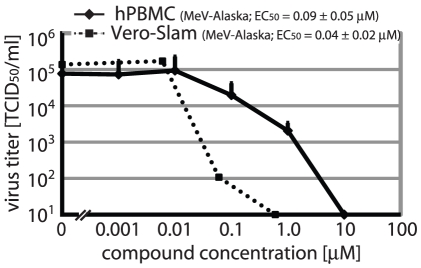
The cellular target range of JMN3-003 extends to primary human cells. Dose-response curves for MeV-Alaska grown in the presence of JMN3-003 on human PBMCs originating from a mixed pool of healthy donors. Vero-Slam cell-based inhibition curves are shown for comparison. Values reflect averages of three replicates. EC_50_ concentrations ± SD are derived from four-parameter non-linear regression modeling.

To further explore whether JMN3-003 meets the profile of a host-directed antiviral, we examined whether the extent of inhibition is determined by the species origin of the host cell used for virus propagation. Based on its broad host cell range, inhibition of influenza A/WSN replication was monitored. In addition to higher mammalian (HT1080 (ATCC CCL-121), HeLa (ATCC CCL-2), MDCK (ATCC CCL-34)) cell lines, cells of rodent (NIH-3T3 (ATCC CRL-1658), MEL B16 (ATCC CRL-6322), BHK-21 (ATCC CCL-10), CHO (ATCC CCL-61)) and avian (DF-1 (ATCC CRL-12203)) origin were tested, which are all permissive for influenza A/WSN infection (table 2). While inhibitory concentrations obtained for all higher mammalian cell lines examined were similar, A/WSN inhibition by JMN3-003 was found inactive on some rodent cell lines and when virus was propagated on murine or avian cells (table 2). However, inhibitory activity extended fully to primary human PBMCs (figure 2). For the latter, inhibition of MeV-Alaska was monitored due to efficient growth of MeV isolates on PBMCs [21]. The host cell species effect of antiviral activity of JMN3-003 is consistent with specific targeting of cellular factors by the compound, while arguing against docking to conserved viral factors or an undesirable promiscuous, unspecific mode of activity.

**Table 2:** Antiviral activity of JMN3-003 is host cell species-specific.

Host Cell		Starting Titer^a^	EC_50_ b	CC_50_ c
		Influenza A/WSN yields in controls	Inhibition of Influenza A/WSN	Metabolic Activity
Cell Line	Origin	[pfu/ml]	[µM]	[µM]
HT1080	Human	1.5×10^6^	0.06±0.002	>75
HeLa	Human	1.6×10^3^	0.02±0.01	>75
MDCK	Canine-dog	3.0×10^7^	0.01±0.08	>75
NIH-3T3	Rodent-mouse	8.4×10^5^	>10	>75
MEL B16	Rodent-mouse	5.3×10^5^	>10	>75
BHK-21	Rodent-Syrian hamster	1.7×10^7^	0.08±0.01	>75
CHO	Rodent-Chinese hamster	1.5×10^5^	0.07±0.01	>75
DF-1	Avian-chicken	1.3×10^6^	>10	>75

a titers of progeny virus grown on the different cell lines in the presence of vehicle (DMSO) only were determined through plaque assays on MDCK cells.

b EC_50_ concentrations were determined based on four parameter non-linear regression models generated for individual dose-response curves.

c Highest concentration assessed 75 µM.

Active concentrations (EC_50_) of JMN3-003 against influenza A/WSN propagated on a variety of different host cell lines.

### JMN3-003 shows high metabolic stability *in vitro*

**Figure 3:**
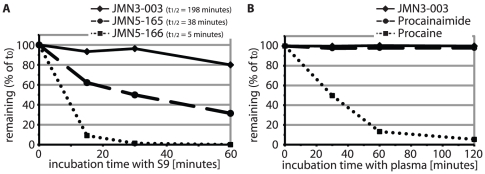
The JMN3-003 scaffold is metabolically stable *in vitro*. **A**) Incubation of the article with human liver S9 fractions for up to 60 minutes, followed by LC-MS/MS analysis of the material remaining. Two analogs of JMN3-003, JMN5-165 and JMN5-166 (figure S1), showed little stability and are included for comparison. Values represent averages of 2 replicates, calculated half-lives (t_1/2_) are given in the figure captures. **B**) Incubation of JMN3-003 for up to 120 minutes with human plasma derived from mixed, healthy donors, followed by LC-MS/MS quantification of the material remaining. Unstable procaine and stable procainamide were examined equally for comparison. Values represent averages of three experiments ± SD.

The central 2-thio-connector found in the chemical scaffold of JMN3-003 may render the compound susceptible to rapid phase I oxidation *in vivo* [22], thus possibly compromising its developmental potential. To test metabolic stability of the substance early in development, we exposed JMN3-003 to human S-9 hepatocyte subcellular fractions as an *in vitro* indicator for phase I metabolism. After a 60-minute exposure, approximately 80% of the input material remained intact, corresponding to an extrapolated half-life of approximately 200 minutes (figure 3A). Unstable analogs of JMN3-003, JMN5-165 and JMN5-166 (figure S1), returned half lives of 38 and 5 minutes in this assay, respectively, confirming metabolic competency of the S9 fractions used.

Assessment of JMN3-003 stability in human plasma in comparison with unstable Procaine and stable Procainamide [23] corroborated these results, since JMN3-003 integrity was virtually unaffected after a 120-minute incubation period (figure 3B). Taken together, these findings suggest desirable metabolic stability for the JMN3-003 scaffold, recommending it for further mechanistic characterization. The data are corroborated by the good metabolic stability reported for the structurally similar compound RDEA-806 (figure S2), a non-nucleoside inhibitor of HIV reverse transcriptase and clinical precedent [24], which shares the 2-thio-connector of JMN3-003 but lacks MeV inhibitory activity in our assays (data not shown).

### Temporary arrest in cell cycle progression

**Figure 4:**
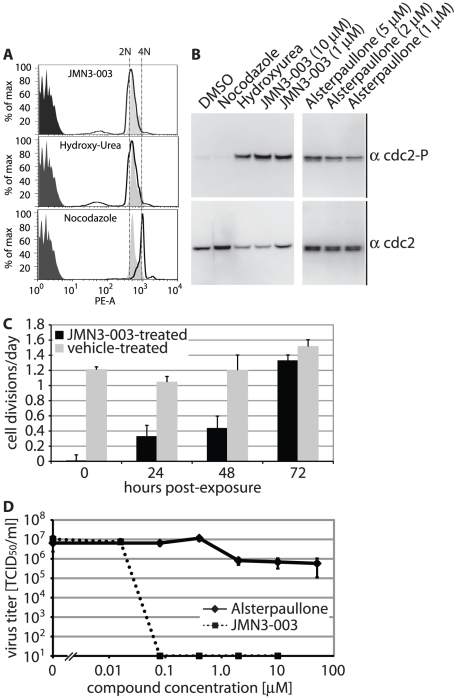
Cell exposure to compound JMN3-003 induces a temporary G_1_/S phase cell cycle arrest. **A**) FACS analysis of acridine orange-stained HeLa cells incubated in the presence of JMN3-003 or hydroxyurea for 36 hours, or nocodazole for 16 hours. Dark grey shaded areas show unstained cells, light grey areas correspond to vehicle-treated control cells, and areas under open black curves represent treated cell populations. Dashed vertical lines indicate 2 N (G_1_/S) and 4N (G_2_/M) DNA contents. Data shown are representative of three experiments and reflect 10,000 events/condition of treatment. **B**) Analysis of the phosphorylation status of cdc2-cyclin B kinase after cell exposure to JMN3-003 through immunoblotting using specific antisera directed against phospho-cdc2 (Tyr15; α cdc2-P) or total cdc2 (α cdc2) for comparison. For control, cells were treated with nocodazole, hydroxyurea, or alsterpaullone (right panel). Results shown are representative of multiple experiments. **C**) Wash-out of JMN3-003 restores cell proliferation. Growth rates of Vero cells were determined after 30-hour exposure of cells to JMN3-003 or vehicle only, followed by wash-out of the substance. Values reflect cell divisions per day and are based on averages of six independent replicate experiments ± SEM. **D**) G_1_/S phase cell cycle arrest does not affect MeV proliferation *per se*. Dose-response curves for alsterpaullone, a nanomolar CDK1/cyclin B kinase inhibitor, and MeV-Alaska grown on Vero-Slam cell. Titers of cell-associated viral particles were determined 36 hours post-infection through TCID_50_ titration. JMN3-003 was examined in parallel for comparison. Values reflect averages of three replicates ± SD.

Since direct cytotoxicity of JMN3-003 was low for all cell lines examined, we next tested the effect of the substance on cell cycle progression. Analysis of the DNA content of cells continuously treated with JMN3-003 for 36 hours by flow cytometry revealed accumulation of cells in a single population with 2N DNA content, which closely resembled the profile of a reference cell population exposed to hydroxyurea but markedly differed from the 4N DNA content of nocodazole-treated cells (figure 4A). Nocodazole interferes with microtubule polymerization [25], resulting in a G_2_/M arrest, whereas hydroxyurea is thought to lead to an arrest in the G_1_/S-phase through depletion of cellular dNTP pools [26], [27]. To further explore the effect of JMN3-003 on cell cycle progression, we monitored the phosphorylation status of the cdc2-cyclin B kinase after exposure of cells to either the compound, hydroxyurea, nocodazole, or alsterpaullone, a nanomolar small molecule inhibitor of cyclin-dependent kinases that reportedly induces a potent G_1_/S-phase cell cycle arrest [28]. Pivotal in regulating the G_2_/M transition, cdc2-cyclin B kinase is inactivated through phosphorylation during the G_2_-phase. Accumulation in its phosphorylated form thus indicates a G_1_ arrest. As in hydroxyurea- and alsterpaullone-treated controls, exposure of cells to JMN3-003 resulted in increased steady state levels of phosphorylated cdc2-cyclin B kinase, supporting a G_1_-phase arrest (figure 4B).

To test whether this JMN3-003-induced arrest is permanent or temporary, we next incubated cells in the presence of compound or vehicle alone for 30 hours, followed by removal of the substance and reseeding of cells at identical densities. Monitoring cell growth over an additional 72-hour incubation period in the absence of JMN3-003 revealed that proliferation rates resumed those of untreated control cells after removal of the compound (figure 4C), indicating reversibility of the growth arrest.

In contrast to members of the orthomyxovirus family, paramyxovirus replication takes place in the cytosol and, thus, is considered not to be immediately dependent on active cell proliferation [1]. In fact, MeV itself has been shown to induce a G_1_/S arrest in infected T lymphoyctes [29], [30], confirming that cell cycle progression is not required for virus replication. To directly test whether the JMN3-003-mediated growth arrest *per se* is causal for the antiviral effect of the compound, we generated MeV-Alaska inhibition curves of JMN3-003 in comparison with the cyclin-dependent kinase inhibitor alsterpaullone. Even at the highest concentration assessed (50 µM), alsterpaullone caused only a marginal reduction in MeV yields (figure 4D). These findings indicate that the antiviral effect of JMN3-003 is based on an upstream effect of the compound rather than being a consequence of the cell cycle arrest itself.

### Cellular mRNA production and protein biosynthesis are unperturbed by JMN3-003

**Figure 5:**
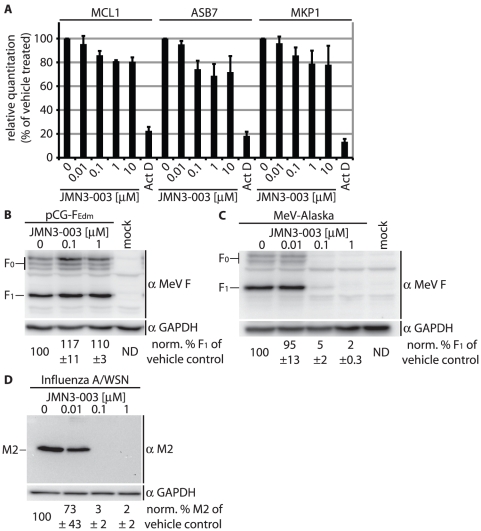
Host cell mRNA synthesis and translation are unaffected by compound JMN3-003. **A**) Relative TaqMan RT-PCR-based quantitation of three unstable cellular mRNAs (MCL1, ASB7, MKP-1) after exposure of cells to JMN3-003 for six hours. Controls were treated with Actinomycine D (Act D) for comparison. C_T_ values are expressed relative to vehicle-treated samples and reflect averages of three independent experiments, each analyzed in triplicate, ± SD. **B–D**) Expression of virus-encoded but not host cell or plasmid-encoded viral proteins is blocked by JMN3-003. Immunodetection of transiently expressed MeV-F (**B**), virus-encoded MeV-F (**C**), and virus-encoded influenza A/WSN M2 (**D**) in cell lysates after incubation of cells in the presence of compound or vehicle only (DMSO) for 30 hours. As internal cellular standard, membranes were probed for GAPDH in parallel. Numbers correspond to average densitometric quantitations ± SD of three experiments, representative immunoblots are shown. (ND: not determined).

To explore whether growth arrest of treated cells coincides with reduced host cell RNA synthesis or overall cell protein biosynthesis, we next assessed the effect of JMN3-003 on host mRNA and protein production. Relative levels of three signature host mRNAs with short half lives, MCL1, ASB7 and MKP1 [31], [32], were determined by real time PCR after incubation of cells in the presence of different JMN3-003 concentrations ranging from 0.01 to 10 µM. In all cases, mRNA levels of JMN3-003-exposed cells were similar to those of the vehicle-treated references, while exposure to Actinomycin D, which blocks RNA synthesis through arrest of the transcription initiation complex [33], resulted in a major reduction in relative mRNA levels (figure 5A).

Immunodetection of cellular GAPDH and plasmid-encoded MeV F protein under the control of the CMV promoter demonstrated that productive transcription in the presence of the compound furthermore coincides with uninterrupted translation and, in the case of F, co-translational insertion into the host secretory system (figure 5B). Furthermore, equivalent levels of proteolytically processed F_1_ material in JMN3-003 and vehicle-exposed cells indicated that intracellular vesicular transport remains intact in the presence of JMN3-003, since cleavage is mediated by the cellular protease furin in a late-Golgi compartment [1]. In contrast to host-encoded or transiently expressed proteins, expression of virus-encoded proteins in the context of paramyxovirus or orthomyxovirus infection was fully blocked by 100 nM JMN3-003 (figures 5C and D). Thus, these observations demonstrate that the compound efficiently suppresses the expression of virus-encoded proteins, but that this is not due to general interference of the inhibitor with cellular mRNA synthesis or translation. This phenotype suggests possible interference of JMN3-003 with early steps of the viral life cycle, such as entry or viral RdRp activity, as the basis for antiviral activity.

### Inhibition of a post-entry step of the viral life cycle

**Figure 6:**
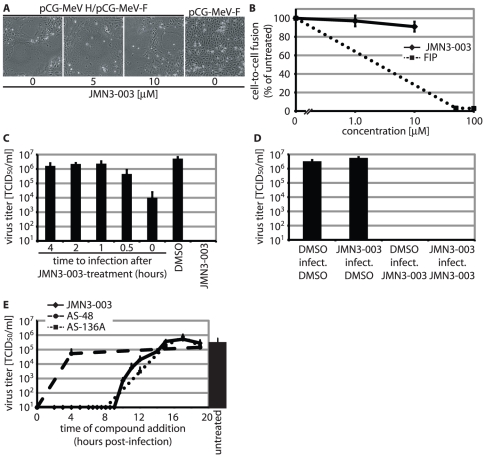
In time-of-addition assays, JMN3-003 shows the inhibition profile of an RdRp blocker. **A–B**) Cell-to-cell fusion is unaffected by the compound. Microphotographs of MeV-H and F expressing Vero-Slam cells (**A**) and quantitative cell-to-cell fusion assays (**B**) show membrane fusion activities in the presence of JMN3-003 similar to those observed for vehicle (DMSO)-treated controls. The effect of fusion inhibitory peptide (FIP) is shown in (B) for comparison. **C**) JMN3-003 antiviral activity is reversible and not based on cell priming. Vero-Slam cells were pre-treated with 1.0 µM JMN3-003 for 60 minutes, followed by compound wash-out and incubation for the indicated time periods; at t_0_, cells were infected with MeV-Alaska. **D**) JMN3-003 lacks virucidal activity. MeV-Alaska particles were incubated with 1.0 µM JMN3-003 for 60 minutes, followed by dilution of compound to 1.0 nM and infection of cells at an MOI of 0.033 in the presence of vehicle (JMN3-003/infect./DMSO). Equally treated controls received vehicle only (DMSO/infect./DMSO), compound only after infection (DMSO/infect./JMN3-003), or compound for the duration of the experiment (JMN3-003/infect./JMN3-003). **E**) Addition of JMN3-003 (1.0 µM final concentration) at the indicated times post-infection of cells with MeV-Alaska. For comparison, inhibition profiles of the MeV entry inhibitor AS-48 (75 µM) and RdRp blocker AS-136A (25 µM) are shown. Controls received vehicle only (DMSO) at the time of infection. For (C–E), values show titers of cell associated viral particles (TCID_50_/ml) and represent averages of at least three experiments ± SD.

To differentiate between those alternatives and identify the point of arrest in the viral life cycle induced by JMN3-003, we first examined whether the compound blocks membrane fusion and thus viral entry. Expression of plasmid-encoded paramyxovirus envelope glycoproteins in receptor-positive cells typically results in extensive cell-to-cell fusion, the hallmark cytopathic effect associated with most paramyxovirus infections *in vitro* [1]. Transient membrane fusion assays allow a quantitative assessment of whether an inhibitor blocks viral entry or post-entry steps of the viral life cycle [20], [34]. When we examined MeV glycoprotein-mediated cell-to-cell fusion microscopically (figure 6A) and in a luciferase reporter-based quantitative cell-to-cell fusion assay (figure 6B) in the presence of JMN3-003, we observed extensive membrane fusion indistinguishable from that seen in vehicle-treated controls, indicating that the compound does not act as an entry inhibitor.

To determine whether JMN3-003 predisposes host cells against viral infection by inducing an antiviral state, we pre-treated cells with the compound, followed by wash-out of the substance and virus infection after different time periods. Independent of incubation time after removal of the compound, we could not detect any substantial inhibitory effect in this set-up (figure 6C), arguing against priming of the innate antiviral response by JMN3-003. Likewise, preincubation of viral particles with JMN3-003 prior to removal of the article and infection lacked any appreciable antiviral effect (figure 6D), excluding direct virucidal activity of the substance.

When added in a time-of-addition experiment at distinct time points post-infection in comparison with two previously characterized, pathogen-targeted antivirals, the inhibition profile of JMN3-003 was distinct from that of the entry inhibitor AS-48 [34] but very closely resembled the profile of the AS-136A RdRp blocker class ([20], figure 6E). Thus, these data point towards inhibition of the viral RdRp activity by JMN3-003 as one possible underlying mechanism for antiviral activity of the compound.

### Host-directed inhibitor of viral RdRp activity

**Figure 7:**
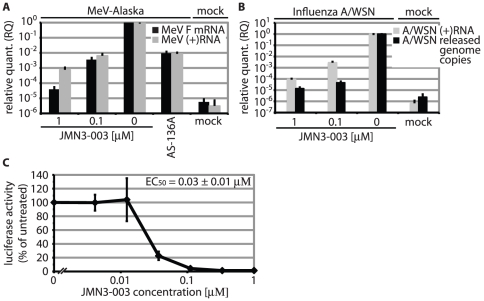
Compound JMN3-003 inhibits viral RNA synthesis. **A**) Relative quantitations of MeV F mRNA and antigenome (+RNA) levels after incubation of infected cells in the presence of compound for 40 hours. Samples were normalized for vehicle only (DMSO)-treated cells and ΔΔC_T_ values calculated using cellular GAPDH as reference. Mock samples remained uninfected. Averages of three independent experiments, assessed in triplicate each, ± SD are shown. **B**) Quantitation of influenza A/WSN segment seven antigenome (+RNA) and of released progeny genomic RNA (genome copies) after incubation of infected MDCK cells in the presence of compound for 24 hours. For +RNA quantitation, samples were normalized and ΔΔC_T_ values calculated as outlined in (A). Released genome copies were quantified by TaqMan RT-PCR relative to an external standard, then normalized for vehicle-treated controls. Averages of four experiments, assessed in triplicate each, ± SD are shown. **C**) Luciferase reporter-based assessment of viral RdRp activity in the presence of JMN3-003. BHK-T7 cells transfected with plasmids encoding the MeV minireplicon reporter system were incubated in the presence of JMN3-003 or vehicle only for 36 hours. Values were normalized for luciferase activities found in vehicle (DMSO)-treated controls and represent averages of three experiments assessed in duplicate each ± SD.

For myxovirus infection, the viral RdRp complex mediates both genome transcription and replication to express viral proteins and generate progeny genomes, respectively. Replication occurs through generation of an antigenome of positive polarity, which then serves as template for negative strand genome synthesis [1]. To directly test whether JMN3-003 affects viral RdRp activity in the context of virus infection, we determined the copy numbers of MeV-Alaska mRNA and antigenome in infected, compound-treated cells relative to vehicle-treated controls by quantitative RT-PCR. Presence of JMN3-003 caused a dose-dependent reduction in viral RNA levels (figure 7A). At a concentration of 100 nM, for instance, we observed a >100-fold reduction of viral mRNA and antigenome copy numbers relative to vehicle-treated samples, indicating potent inhibition of viral replication. For comparison, a concentration of 25 µM of the RdRp inhibitor AS-136A, a nanomolar blocker of MeV replication [35], was required to achieve comparable mRNA and antigenome reduction levels (figure 7A).

When this assay was applied to orthomyxovirus infection, we likewise observed a dose-dependent inhibition of influenza A/WSN antigenome levels relative to vehicle treated controls (figure 7B). Parallel quantification of genome copy numbers of released progeny virus demonstrated that an approximate >100-fold drop in relative viral antigenome levels correlates to a >10,000-fold reduction in genome copies of released progeny virions (figure 7B).

Assessment of viral RdRp activity in a plasmid-based minireplicon reporter system confirmed dose-dependent inhibition of RdRp by JMN3-003 also in a sub-infection setting, since luciferase reporter expression was fully blocked at compound concentrations of approximately 100 nM (figure 7C). Taken together, these data suggest indirect inhibition of the viral polymerase complex through interaction of the compound with a cellular cofactor required for RdRp activity as the basis for the antiviral effect of JMN3-003.

### JMN3-003 does not induce rapid emergence of viral resistance

**Figure 8:**
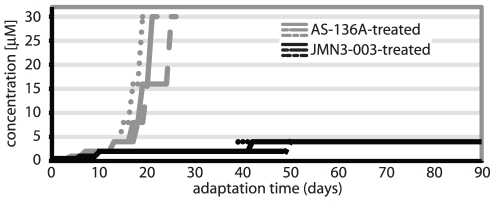
JMN3-003 prohibits rapid emergence of viral resistance *in vitro*. MeV-Alaska remains sensitive to the compound after continued adaptation events for a 90-day period, while resistance (extensive viral CPE detectable in the presence of 30 µM compound) to pathogen-directed AS-136A emerges in step-wise adaptations after 15–25 days. Three independent adaptations (represented by solid, dotted and dashed lines, respectively) were pursued for each compound.

It has been suggested for different viral pathogens that a host-directed antiviral approach has the potential to reduce the frequency of viral escape from inhibition compared to direct targeting of pathogen components [14], [15]. To explore whether resistance to JMN3-003 could be induced experimentally, we attempted stepwise viral adaptation to growth in the presence of the compound in comparison with the pathogen-specific MeV RdRp inhibitor AS-136A [36]. Following an escalating dose scheme, inhibitor concentrations were doubled when virus-induced cytopathicity became detectable microscopically. While robust resistance to the pathogen-targeted AS-136A control emerged rapidly in an approximate 15 to 20-day time window (tolerated dose at the end of adaptation was ≥30 µM, equivalent to ≥100-fold resistance), only marginal increases in the tolerated dose could be detected for JMN3-003 after 90 days of continued viral incubation in the presence of the substance (figure 8). These results are consistent with a host-directed mechanism of action of JMN3-003 and suggest the existence of a systemic barrier that prevents rapid viral escape from inhibition by the article.

## Discussion

In recent years, host cell-directed antivirals have experienced growing recognition as a new concept for the development of advanced generation antivirals with the potential to counteract the challenge of preexisting or rapidly emerging viral resistance [14], [15]. Novel automated genomics and proteomics analyses have greatly advanced our insight into host-pathogen interactions [37], [38], [39], [40], [41], [42], [43], [44]. These studies have underscored the notion that several cellular pathways are exploited for virus replication [45], [46], supporting the hypothesis that a host-directed antiviral may enjoy an expanded viral target range, rendering it effective for the treatment of several related viral diseases.

Technologies applied for host-directed drug discovery include cDNA and siRNA-based microarray analyses combined with pathway-guided data mining [47], [48], [49], [50], [51], loss-of-function screens using aptamers or small oligonucleotides [52], [53], [54], [55], [56], [57], [58], protein array analyses [59] and chemical library screening [60], [61]. By combining automated library screening [19] with counter screens against a variety of related viral pathogens of the myxovirus families, we have identified a candidate scaffold that, after moderate hit-to-lead chemistry, adheres to the profile of a host-directed antiviral based on several lines of evidence: I) antiviral activity is host cell species-dependent, indicating specific interaction with a distinct host factor rather than a viral component. Host cell-specific activity is incompatible with compound docking to conserved viral factors. For example, carbohydrate structures exposed on viral envelope glycoproteins that are targeted by antiviral lectins such as pradimicin A [62]. Furthermore, it is incompatible with an undesirable unspecific, promiscuous mode of action of the compound [63]; II) affinities against a panel of human pathogens of the paramyxovirus family as well as laboratory adapted and wild type influenza virus isolates were very similar throughout (average EC_50_ concentrations are ∼40 nM). Equivalent active concentrations argue against compound docking to distinct viral components and suggest that inhibition of distinct myxovirus families follows the same mechanism of action; III) *in vitro* adaptation attempts to induce viral resistance were unsuccessful even after extended exposure times to the drug. A full assessment of the frequency of viral escape from inhibition by JMN3-003 will certainly need to include *in vivo* virus adaptation attempts in suitable animal models, since the rate of resistance build-up may vary between tissue culture and *in vivo* settings. We nevertheless reliably induced resistance in less than 30 days to a pathogen-directed MeV RdRp inhibitor that was analyzed in parallel, which is fully consistent with our previous experience [36] and provides confidence for the validity of our overall experimental design for viral adaptation.

Mechanistic analysis of the bioactivity of the JMN3-003 compound class through characterization of exposed cells and time-of-addition experiments revealed two distinct phenotypes, a temporary cell cycle arrest in the G_1_/S phase and an arrest in the myxovirus life cycle at a post-entry step. Current libraries of chemical analogs of JMN3-003 do not yet permit a definitive conclusion as to whether both activities adhere to discrete structure-activity relationships or are causally linked, but a bulk of experimental data demonstrate that host cell cycle arrest *per se* has no inhibitory effect on replication of paramyxoviruses such as MeV. Not only does the virus itself induce a G_1_/S-phase arrest in infected T lymphocytes [29], [30], we also found that exposure of infected cells to alsterpaullone, a potent blocker of G_1_/S-phase cell cycle progression through nanomolar inhibition of cellular cyclin-dependent kinases [28], did not affect the extent of virus replication even at concentrations exceeding reported alsterpaullone EC_50_ values by more than 1,000-fold. Likewise consistent with the notion that the antiviral activity of JMN3-003 is not based on cell cycle arrest itself, virus inhibition was not restricted to the context of immortalized, rapidly dividing tissue culture cell lines but extended with equal potency to primary human PBMCs.

Reversible cell cycle arrest and block of virus replication indicate non-covalent docking of JMN3-003 to its target structures, which is corroborated by the compound's stability, low chemical reactivity profile and the complete absence of virucidal activity in pre-incubation settings. An inhibition profile of JMN3-003 closely mimicking that of AS-136A, the pathogen-directed blocker of MeV RdRp targeting the viral L polymerase protein [36], and the block in viral RdRp activity in the context of viral infection and minireplicon reporter assays by JMN3-003 consistently point towards interaction of the compound with a host cofactor essential for RdRp function as the basis for its antiviral activity. While viral RdRp depends on a variety of host cell components [1], unperturbed cellular mRNA synthesis and, thus, uninterrupted host RNA polymerase function in the presence of compound exclude interference of JMN3-003 with essential transcription initiation factors.

Recently, accumulating evidence has implicated host cell kinases as regulators of the activity of RdRp complexes of different negative-strand RNA viruses [64]: host cell kinases of the PI3K-Akt pathway manipulate paramyxovirus RdRp activity through Akt-mediated phosphorylation of the viral phosphoprotein, an essential component of the RdRp complex. Furthermore, Akt activity itself is upregulated through activation of PI3K during influenza A infection via direct interaction of the viral NS1 protein with PI3K [65], [66]. In the case of MeV, however, published data [67], [68] and our own observations (Krumm and Plemper, unpublished) demonstrate that Akt inhibition causes a moderate reduction in virus release, whereas titers of cell-associated progeny particles remain unchanged. While this rules out the PI3K-Akt pathway as a direct target for JMN3-003, it illuminates the intricate regulatory interactions between pathogen and host, which provide a wealth of possible points of entry for antiviral intervention. Future identification of the molecular target of JMN3-003 carries high potential to further our understanding of these interactions and may conceivably provide a basis for pharmacophore extraction and structure-driven scaffold optimization.

We note that the central sulfur in the JMN3-003 chemical scaffold could potentially render the molecule vulnerable to rapid phase I oxidation and thus compromise both metabolic stability and bioavailability. For instance, it has been reported that flavin-containing monooxygenases [69], dioxygenases [70] and cytochrome P-450 enzymes [71] catalyze oxidation of alkylaryl sulfides to sulfoxides (R_2_S = O). However, the high stability of JMN3-003 in the presence of human hepatocyte subcellular fractions and human plasma argues against an undesirable short *in vivo* half-life of the substance. This is corroborated by good metabolic stability of the structurally similar HIV reverse transcriptase inhibitor RDEA-806 [72], [73], which shares the central 2-thio-acetamide connector with JMN3-003 and has achieved success in clinical trials: the compound was well tolerated in both Phase 1 and 2a studies after single or multiple oral doses and showed no drug-related CNS toxicity [72], [73], creating a clinical precedence for the applicability of the broader scaffold. Although RDEA-806 follows a different mechanism of action than JMN3-003 and lacks any anti-paramyxovirus activity, the structural similarities provide sufficient confidence for the overall developmental potential of the JMN3-003 class to recommend it as a promising candidate for advanced synthetic optimization towards preclinical validation and development.

*In toto*, we have identified a novel chemical class of viral inhibitors that block viral RdRp activity with a host factor-mediated profile. A complete activity workup after synthetic identification of a clinical lead analog will be required to fully appreciate the range of the different viral families inhibited by the substance. However, we consider human pathogens of the myxovirus families that are primarily associated with acute disease among the most suitable for host-directed antiviral efforts due to anticipated short treatment regimens. While we cannot exclude that resistance to JMN3-003 may eventually emerge in *in vivo* settings, our *in vitro* adaptation efforts support the hypothesis that the mechanism of action of this compound class establishes a strong barrier against rapid viral escape from inhibition.

## Materials and Methods

### Cells and viruses

All cell lines were maintained at 37°C and 5% CO_2_ in Dulbecco's modified Eagle's medium supplemented with 10% fetal bovine serum. Vero (African green monkey kidney epithelial) cells (ATCC CCL-81) stably expressing human signaling lymphocytic activation molecule (CD150w/SLAM), called in this study Vero-SLAM cells [74], and baby hamster kidney (BHK-21) cells stably expressing T7 polymerase (BSR-T7/5 (BHK-T7) cells [75]) were incubated at every third passage in the presence of G-418 (Geneticin) at a concentration of 100 µg/ml. Lipofectamine 2000 (Invitrogen) was used for cell transfections. Peripheral blood mononuclear cells (PBMCs) were prepared through overlay of whole blood samples from mixed, healthy human donors (Emory University Institutional Review Board approval IRB00045690, Phlebotomy of Healthy Adults for Research in Infectious Diseases and Immunology) on Ficoll Hypaque solution, followed by centrifugation at 240×g for 30 minutes at room temperature and removal of the interphase material. Red blood cells were lysed with RBC lysis solution (Sigma), followed by repeated washing of extracted PBMCs with phosphate buffered saline and transfer to tissue culture plates pre-coated with poly-L-lysine (Sigma). Other primary human cell lines were obtained from PromoCell, Germany. Virus strains used in this study were MeV isolate MVi/Alaska.USA/16.00, genotype H2 (MeV-Alaska) [76], HPIV3, MuV strain South Africa, RSV strain Long, laboratory adapted influenza A strains WSN (H1N1) and PR8/34 (H1N1), swine-origin influenza virus isolates S-OIV Texas and Mexico, vaccinia virus and sindbis virus. To prepare virus stocks, cells permissive for the virus to be amplified (Vero-Slam, Vero, HepG2 (ATCC HB-8065), and Madin-Darby canine kidney (MDCK)) were infected and incubated at 37°C. Cell-associated paramyxovirus and vaccinia virus particles were harvested by scraping cells in OPTIMEM (Invitrogen), followed by release of virus through two consecutive freeze-thaw cycles. Influenza virus and sindbis virus particles were harvested from cell culture supernatants. Titers of MeV and MuV were determined through 50% tissue culture infective dose (TCID_50_) titration according to the Spearman-Karber method [77] as described [78], titer of all other viruses were determined by plaque assay on permissive cells.

### Influenza A titration by TaqMan RT-PCR

To determine genome copy numbers of released progeny influenza A particles (strains WSN, PR8/34, S-OIV Texas and Mexico), culture supernatants of infected MDCK cells (4×10^5^ cells/well in a 12-well plate format) were harvested and total RNA prepared using a QIAcube automated extractor and the QIAamp viral RNA mini kit reagent. Purified RNA was then subjected to quantitative real time (qRT) PCR analysis using an Applied Biosystems 7500 Fast real-time PCR system and the qRT-PCR TaqMan Fast Virus 1-Step Master Mix (Applied Biosystems). Primers and probe are based on recent reports [79] and universally reactive with all influenza A strains including the recent S-OIV (H1N1) isolates. To generate a qRT-PCR standard, genome segment seven of influenza A/WSN was subcloned into pCR2.1-TOPO vector (Invitrogen) and copy numbers of the resulting standard calculated using Promega's BioMath Calculator tools (http://www.promega.com/biomath/). For each TaqMan reaction, 10-fold serial dilutions of the linearized plasmid ranging from 10^7^ to 10^1^ were amplified in parallel.

### Compound synthesis

Chemical synthesis of compounds AS-48, AS-136A and RDEA-806 was achieved as previously described [24], [34], [36]. Synthesis of JMN3-003, N-(4-methoxyphenyl)-2-nitroaniline (substance (3) in figure S1), and analogs JMN5-165 and JMN5-166 was achieved as outlined schematically in figure S1. To prepare inhibitor stocks, compounds were dissolved at 75 mM in DMSO.

### Viral CPE-reduction assay

Vero-SLAM cells were infected with MeV-Alaska at an MOI of 0.4 pfu/cell in the presence of the inhibitor analyzed ranging from 75 µM to 293 nM in two-fold dilutions. At 96 hours post-infection, cell monolayers were subjected to crystal violet staining (0.1% crystal violet in 20% ethanol), and the absorbance of dried plates at 560 nm determined. Virus-induced cytopathicity was then calculated according to the formula [% rel. CPE = 100−(experimental-minimum)/(maximum-minimum)*100], with minimum referring to infected, vehicle-treated wells and maximum to mock-infected wells.

### Virus yield reduction assay

Cells were infected with the specified myxovirus at an MOI = 0.1 pfu/cell (all paramyxoviruses assessed), 0.05 pfu/cells (influenza viruses), 1.0 (vaccinia virus), or 10 sindbis virus) in the presence of a range of compound concentrations or equivalent volumes of solvent (DMSO) only, and incubated in the presence of compound at 37°C. When vehicle treated controls approached the end of the logarithmical growth phase, progeny viral particles were harvested and titered by TCID_50_ titration, plaque assay or TaqMan real-time PCR, respectively, as described above. Plotting virus titers as a function of compound concentration allowed quantitative assessment of resistance. Where applicable, 50% inhibitory concentrations were calculated using the variable slope (four parameters) non-linear regression-fitting algorithm embedded in the Prism 5 software package (GraphPad Software).

### Quantification of compound cytotoxicity

A non-radioactive cytotoxicity assay (CytoTox 96 Non-Radioactive Cytotoxicity Assay, Promega) was employed to determine the metabolic activity of cell after exposure to the compound. In a 96-well plate format, 10,000 cells per well were incubated at 37°C for 24 hours in four replicates per concentration tested in the presence of compound in two-fold dilutions starting at 75 µM. Substrate was then added and color development measured at 490 nm using a BioRad plate reader. Values were calculated according to the formula [% toxicity = 100−((experimental-background)/(maximum(vehicle treated)-background)*100)]. Values were plotted in dose-response curves and, if applicable, CC_50_ concentrations calculated.

### *In vitro* assessment of metabolic and plasma stability

JMN3-003 was mixed with liver S9 fractions (protein concentration 2.5 mg/ml) from pooled mixed gender humans (XenoTech) at a final concentration of 1 µM and reactions initiated by the addition of cofactors (1.14 mM NADPH, 1.43 mM glucose-6-phosphate, 1.43 mM uridine 5′-diphosphoglucuronic acid, 9.42 mM potassium chloride, 2.28 mM magnesium chloride) in 100 mM potassium phosphate buffer, pH 7.4. Samples were incubated at 37°C with mixing, aliquots removed after 0, 15, 30 and 60 minutes and subjected to reversed-phase LC-MS/MS (Applied Biosystems API 4000 QTRAP with heated nebulizer; Turbo IonSpray for JMN5-166) analysis. Peak areas were measured to calculate half life and percent of input compound remaining according to the formulas t_1/2_ = (−0.693/slope of linear regression analysis of log transformed peak area versus) and % input remaining = (peak area of test compound at t_x_/peak area of test compound at t_0_)*100. Positive controls to assess the metabolic competency of the liver S9 fractions were 7-Ethoxycoumarin, Propranolol, and Verapamil (Sigma), which were analyzed in parallel to the article. To determine compound plasma stability, articles were mixed with freshly prepared human plasma at a final concentration of 0.5 mM and incubated at 37°C for up to 120 minutes. Aliquots were removed at distinct time points as indicated and analyzed by LC-MS/MS with detection of the compound at 254 nm. Values are expressed as percent of compound remaining at each time relative to the amount of that compound present at the starting time point.

### Flow-cytometric analysis of cell cycle progression

Actively proliferating HeLa cells were exposed to JMN3-003 (10 µM), hydroxyurea (4 mM), or nocodazole (200 ng/ml) for 36 hours, followed by resuspension in buffer I (20 mM citrate/PO, pH 3.0, 0.1 mM EDTA, 0.2 M Sucrose, 0.1% Triton X-100) and staining in buffer II (10 mM Citrate/PO, pH 3.8, 0.1 M sodium chloride, 20 µg/ml acridine orange) as described [80]. Green fluorescence at 525 nm resulting from DNA intercalating acridine orange was then measured using a BD LSRII flow cytometer and FlowJo software (Tree Star) for data analysis. For comparison, unstained and stained, solvent-only exposed cells were examined in parallel.

### SDS-PAGE and immunoblotting

Cells were lysed with RIPA buffer (50 mM Tris/CL, pH 7.2, 1% deoxycholate, 0.15% sodium dodecylsulfate, 150 mM sodium chloride, 50 mM sodium fluoride, 10 mM EDTA, 1% NP-40, 1 mM PMSF, protease inhibitors). Aliquots with equal total concentrations of cleared lysates (20,000×g; 10 min; 4°C) were mixed with 2x-urea buffer (200 mM Tris, pH 6.8; 8 M urea; 5% sodium dodecyl sulfate (SDS); 0.1 mM EDTA; 0.03% bromphenol blue; 1.5% dithiothreitol) and denatured for 25 min at 50°C. Samples were then fractionated on 10% SDS-polyacrylamide gels, blotted to polyvinylidene difluoride (PVDF) membranes (Millipore) and subjected to enhanced chemiluminescence detection (Pierce) using specific antisera directed against phosphorylated or non-phosphorylated cdc2-cyclin B kinase (Cell Signaling Technology), GAPDH (Abcam), the cytosolic tail of the MeV F protein [81], or influenza A/WSN virus M2 (Thermo Scientific). Immunostained PVDF membranes were developed using a ChemiDoc XRS digital imaging system (Bio-Rad) and horseradish peroxidase conjugated anti-species IgG (mouse or rabbit) antibodies. For densitometry, signals were quantified using the QuantityOne software package (Bio-Rad).

### Assessment of cell growth rates

Vero cells were seeded at a density of 6×10^5^ cells and incubated in the presence of 10 µM JMN3-003 or vehicle only for 30 hours at 37°C. Cells were then washed extensively and reseeded at a density of 1×10^5^ cells per well, followed by continued incubation at 37°C and assessment of life/dead cell numbers every 24 hours using a Countess automated cell counter (Invitrogen). Cells were reseeded as before when fastest growing cultures approached confluency. Growth rates were calculated for each 24-hour time interval using the Prism software package (GraphPad Software Inc.) based on the formula Y = Y_0_*exp(K*X) with Y equaling life cell numbers, Y_0_ the Y value at the starting time (t_0_), and K the growth constant equaling ln(2)/doubling-time.

### Quantification of cellular and viral mRNA levels

Cells were infected with either recombinant MeV Edmonston (recMeV-Edm) [82] (Vero cells, MOI = 1.0) or influenza A/WSN (MDCK cells, MOI = 0.05), followed by removal of inocula one hour post-infection and addition of JMN3-003 in growth media at 0.1 µM or 1 µM. All MeV infected wells received in addition fusion inhibitory peptide (FIP, Bachem) at 100 µM to prevent premature breakdown of the monolayer through viral CPE in the vehicle control wells prior to RNA extraction. Twenty-four (influenza A/WSN) or forty (recMeV-Edm) hours post-infection, total RNA was prepared from all wells using the QIAcube automated extractor and the RNeasy Mini Kit (Qiagen), and subjected to reverse transcription using Superscript II Reverse Transcriptase (Invitrogen). For RNA samples originating from recMeV-Edm infected cells, antigenome-specific primer 5-GGCTCCCTCTGGTTGT or oligo-dT primer (viral mRNA and GAPDH quantification) were used for cDNA priming. In the case of samples originating from influenza A/WSN infected cells, primers for cDNA synthesis were 5-AGTAGAAACAAGGTAGTTT (antigenome) or oligo-dT (mRNA and canine GAPDH). Real-time reactions were carried out using an Applied Biosystems 7500 Fast real-time PCR *system and iQ* Fast SYBR Green Supermix with ROX (Bio-Rad). Probes were a fragment at the N/P junction (MeV antigenomic RNA, 5-AACCAGGTCCACACAG and 5-GTTG TCTGATATTTCTGAC), a fragment of MeV F mRNA (5-GTCCACCATGGGTCTCAAGGTGAACGTCTC and 5-CAGTTATTGAGGAGAGTT), a fragment of human GAPDH (SABiosciences proprietary primers), a fragment of influenza A/WSN segment seven (influenza A/WSN antigenomic RNA, 5-tagctccagtgctggtct and 5-AAGGCCCTCCTTTCAGTCC), and a fragment of canine GAPDH (Qiagen proprietary primer). Melting curves were generated at the end of each reaction to verify amplification of a single product. To calculate ΔΔC_T_ values, CT values obtained for each sample were normalized for GAPDH as reference and then ΔC_T_ values of JMN3-003-treated samples normalized for the FIP-treated controls. Final quantification was based on three independent experiments in which each treatment condition and RT primer setting were assessed in triplicate. To assess the relative quantities of cellular mRNA, 9×10^5^ HeLa cells were incubated in the presence of JMN3-003 (0.01, 0.1, 1.0, 10.0 µM final concentration), AS-136A (25 µM), Actinomycine D (5 µg/µl), or vehicle only for six hours at 37°C, followed by preparation of total RNA as described above. Quantitative TaqMan RT-PCR was again achieved using the TaqMan Fast Master Mix (Applied Biosystems) combined with proprietary primer and probe sets specific for Induced myeloid leukemia cell differentiation protein 1- (MCL1), MAPK phosphatase 1 (MKP1), and ankyrin repeat and SOCS box-containing protein 7- (ASB7) encoding mRNAs (Applied Biosystems). Samples were standardized for GAPDH as before and normalized values expressed relative to the equally analyzed vehicle-treated controls.

### Quantitative cell-to-cell fusion assays

An effector cell population (3×10^5^ cells/well) was cotransfected with 2 µg each of MeV H and F expression plasmids. To inhibit fusion until the cell overlay, the effector cells are incubated in the presence of 100 µM fusion inhibitory peptide (Bachem). Single transfections of plasmids encoding MeV F served as controls. Target cells (6×10^5^ cells/well) were transfected with 4 µg of the reporter plasmid encoding firefly luciferase under the control of the T7 promoter. Two hours post-transfection, modified vaccinia virus Ankara expressing T7 polymerase at an MOI of 1.0 PFU/cell was added to the effector cells. Following incubation for 16 h at 37°C, target cells were detached and overlaid on washed effector cells at a 1∶1 ratio and incubated at 37°C in the presence of different JMN3-003 concentrations as indicated. Four hours post-overlay, cells were lysed using Bright Glo lysis buffer (Promega), and the luciferase activity determined in a luminescence counter (PerkinElmer) after addition of Britelite substrate (PerkinElmer). The instrument's arbitrary values were analyzed by subtracting the relative background provided by values of the controls, and these values were normalized against the reference constructs indicated in the figure legends. On average, background values were <1% of the values obtained for reference constructs. For qualitative assessment, transfected Vero-SLAM cells were photographed 18 hours post-transfection at a magnification of ×200.

### Time of compound addition analysis

For virus pre-incubation assays, 10^7^ infectious MeV-Alaska particles were incubated for 60 minutes at 37°C in the presence of JMN3-003 (1.0 µM final concentration) or vehicle only, followed by 1,000-fold dilution in growth media and transferred to 3×10^5^ Vero-Slam cells/well (corresponding to final compound concentrations after pre-incubation of 1 nM and an MOI = 0.033). Reference wells were kept at 1.0 µM JMN3-003 for the duration of the experiment. Cell-associated viral particles were harvested 24 hours post-infection and infectious titers determined by TCID_50_ titration. To assess cell priming, Vero-Slam cells (3×10^5^/well) were incubated in the presence of JMN3-003 at 1.0 µM for one hour at 37°C at the indicated time points pre-infection, followed by washing and further incubation in growth media. Immediately before infection, cells were reseeded at a density of 2.5×10^5^ per well and infected with MeV-Alaska at an MOI = 0.2 pfu/cell. Inocula were replaced with growth media four hours post-infection and cells incubated for approximately 20 hours. Cell-associated viral particles were then harvested and infectious titers determined by TCID_50_ titration. For post-infection time-of-addition studies, 3×10^5^ Vero-Slam cells/well were infected with MeV-Alaska as before, followed by addition of JMN3-003 (1.0 µM final concentration), entry inhibitor AS-48 (75 µM), or RdRp inhibitor AS-136A (25 µM). Controls received vehicle only. All wells were harvested 19 hours post-infection and titers of cell-associated progeny virus determined by TCID_50_ titration.

### Minireplicon assays

BSR T7/5 cells (5×10^5^/well) were transfected with plasmid DNAs encoding MeV-L (0.24 µg), MeV-N (0.94 µg) or MeV-P (0.29 µg) and 2 µg of the MeV luciferase minigenome reporter plasmid [83]. Control wells included identical amounts of reporter and helper plasmids but lacked the L-encoding plasmid. At the time of transfection, JMN3-003 was added as specified, while control wells received vehicle only for comparison. Thirty-six hours post-transfection, cells were lysed with Bright GLO lysis buffer and relative luciferase activities determined using the Britelite substrate and a luminescence counter as outlined above.

### *In vitro* virus adaptation

Adaptations were carried out essentially as we have previously described [36]. Briefly, Vero-SLAM cells were infected with MeV-Alaska at an MOI of 0.1 pfu/ml and incubated in the presence of gradually increasing JMN3-003 concentrations starting at 0.5 µM. Equally infected cells treated with the virus polymerase targeted RdRp inhibitor AS-136A were examined in parallel. When cultures became over confluent, cells were reseeded for continued incubation in the presence of the same compound concentration as before. At detection of extensive cell-to-cell fusion, cell-associated viral particles were harvested, diluted 10-fold and used for parallel infections of fresh cell monolayers in the presence of compound at unchanged and doubled concentrations. Cultures treated with the highest compound concentrations in which virus-induced cytopathicity became detectable were used for further adaptation. The approach was terminated after 90 days of continued incubation or when virus-induced cytopathicity was readily detectable in the presence of 30 µM compound in accordance with previous results [36].

## Supporting Information

Figure S1 Synthesis of JMN3-003, JMN5-165 and JMN5-166.
(PDF)

Figure S2 Structure of RDEA-806.
(DOCX)
