# Supplementary material for: Potent Host-Directed Small-Molecule Inhibitors of Myxovirus RNA-Dependent RNA-Polymerases
Source: PLoS One. 2011 May 16;6(5):e20069. doi: 10.1371/journal.pone.0020069 (PMC3095640; doi:10.1371/journal.pone.0020069)
Supplement: Figure S1 — Synthesis of JMN3-003, JMN5-165 and JMN5-166. (PDF) [file pone.0020069.s001.pdf]

Figure S1: Synthesis of JMN3-003, JMN5-165 and JMN5-166.

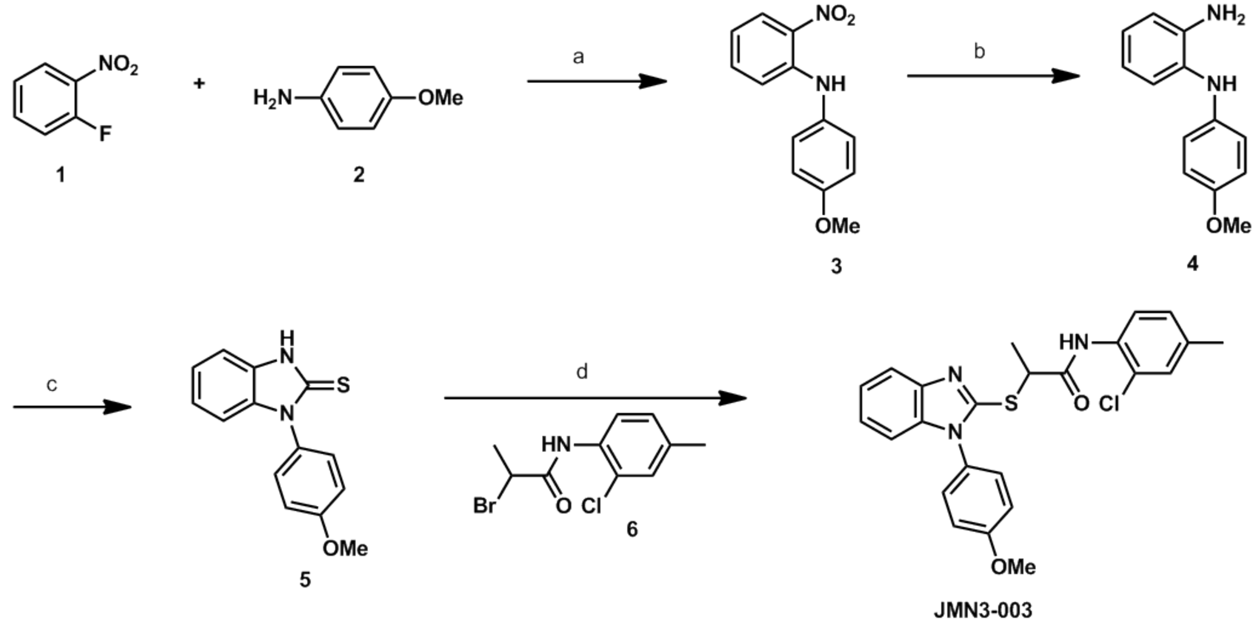

a) K_2CO_3, 160 °C or Et_3N, 150 °C, microwave b) H_2, Pd/C, ethanol, 40 psi c) 1,1'-Thiocarbonyldiimidazole, CH_2Cl_2
d) KO^tBu, ethanol

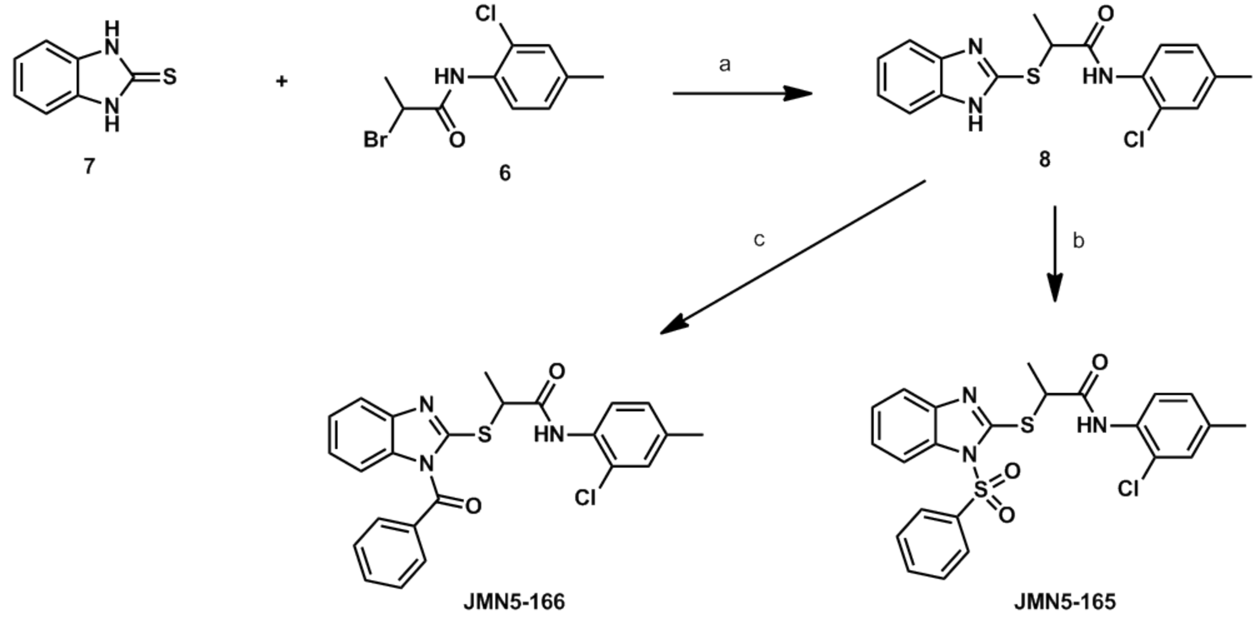

a) NaH, THF b) benzenesulfonyl chloride, *i*-Pr_2NEt, CH_2Cl_2 c) Benzoyl chloride, *i*-Pr_2NEt, CH_2Cl_2

P-anisidine (4.36g, 35.4 mmol), 1-fluoro-2-nitrobenzene (3.8 ml, 36.0 mmol) and Et_3N (4.93 ml, 35.4 mmol) were mixed in a microwave tube and heated at 150°C for one hour. The mixture was stirred in water, ether added and the precipitate filtered. The reddish brown solid was washed with hexanes and dried under vacuum to give 8.0 g of substance (3) in 94% yield. ^1H NMR (400 MHz, $CDCl_3$, δ = 7.24 ppm); δ [illegible] 87[illegible]s, 1H), 10.01 (s, 1H), 8.18 (dd, *J* = 8.0, 1.6 Hz, 1H), 7.33-6.68 (m, 7H), 3.83 (s, 3H). For synthesis of N-(4-methoxyphenyl)benzene-1,2-diamine (substance (4) in supplement 1), 10% Pd/C (300.0 mg, 0.282 mmol) was added to a

suspension of (3) (2.6g, 10.65 mmol) in methanol (40.0 ml) and the mixture exposed to hydrogen at 40 psi for three hours. The mixture was filtered through a pad of celite and the filtrate concentrated. The resultant reddish brown oil was dried under vacuum to give a brown solid in quantitative yield. ^{1}H NMR (400 MHz, $CDCl_3$, δ = 7.24 ppm); δ 9□39□□s□□□H□□□8□□9□8□□6 □m□□□H□□□7□33□7□28□□m□□□H□□□7□2□□7□□6□□m□□2H□□□7□□□6□92□□m□□ 3H□□□ 6□7□□□t, *J* = 8.0 Hz, 1H), 3.83 (s, 3H). To synthesize 1-(4-methoxyphenyl)-1H-benzoimidazole-2-thiol (substance (5) in supplement 1), a solution of substance (4) (7.0g, 32.7 mmol) in CH_2Cl_2 (100.0 ml) at 0°C was combined with 1,1-thiocarbonyldiimidazole (5.83g, 35.9 mmol) and the mixture stirred for 30 minutes. The cold-bath was removed and the mixture stirred for a further two hours before adding water (50 ml). The organic layer was separated and washed with water (50.0 ml), dried over Na_2SO_4, filtered and concentrated. The residue was triturated with CH_2Cl_2 and hexanes to give a grey solid (6.5 g 88% yield). ^{1}H NMR (400 MHz, $CDCl_3$, δ = 7.24 ppm); δ 10.7 (s, 1H), 7.46-7.42 (m, 2H), 7.28-7.09 (m, 5H), 6.95(d, *J* = 7.6 Hz, 1H), 3.93 (s, 3H).

To generate 2-bromo-N-(2-chloro-4-methylphenyl)propanamide (substance (6) in supplement 1), a solution of 2-chloro-4-methyl-aniline (3.0g, 24.8 mmol) in CH_2Cl_2 (100.0 ml) at 0°C was treated with *i*-Pr_2NEt (6.5 ml, 37.3 mmol) and 2-bromo propionyl bromide (3.3 ml, 29.6 mmol) and the mixture stirred for one hour. The organic layer was separated and washed with 1N NaOH (2 x 30.0 ml), dried over Na_2SO_4, filtered and concentrated to give a white solid (5.1g, 74% yield) that was dried under vacuum and used without further purification. ^{1}H NMR (400 MHz, $CDCl_3$, δ = 7.24 ppm); δ□8.60 (s, 1H), 8.18 (d, *J* = 8.4 Hz, 1H), 7.22 (s, 1H), 7.09 (d, *J* = 8.0 Hz, 1H), 4.59 (q, *J* = 7.2 Hz), 2.32 (s, 3H), 1.99 (d, *J* = 7.2 Hz, 3H). To obtain JMN3-003 as the final product, a suspension of substance (5) (300.0 mg, 1.17 mmol) in ethanol (10.0 ml) was combined with KO^tBu (150.0 mg, 1.34 mmol) and the mixture stirred at room temperature for one hour. To the reaction was added substance (6) (340.0 mg, 1.23 mmol) and the mixture stirred for five hours. Concentrated NH_4Cl (10.0 ml) and dichloromethane (10.0 ml) were added to the reaction, the organic layer separated and washed with brine (10.0 ml), then dried over Na_2SO_4, filtered and concentrated. The crude product was purified by silica column chromotography (hexanes/ethylacetate) to give JMN3-003 (420.0 mg, 80% yield) as a white solid. ^{1}H NMR (400 MHz, $CDCl_3$, δ = 7.24 ppm); δ 10.45 (s, 1H), 8.22 (d, *J* = 8.4 Hz, 1H), 7.34-7.0 (m, 9H), 4.91 (q, *J* = 7.6 Hz, 1H), 3.88 (s, 3H), 2.24 (s, 3H) 1.6 (d, *J* = 7.2 Hz, 3H).

To synthesize 2-(1H-benzoimidazol-2-yl)thio-N-(2-chloro-4-methylphenyl) propanamide (substance (8) in supplement 1), a solution of 2-mercaptobenzimidazole (500.0 mg, 3.33 mmol) in tetrahydrofuran (30.0 ml) was treated with NaH (150.0 mg, 3.75 mmol) and the mixture stirred for 10 minutes. Substance (6) (supplement 1) (920.0 mg, 3.33 mmol) was added to the reaction and the mixture stirred for six hours. Water (15 ml) was added and the product extracted using ethylacetate, the organic layer then dried over Na_2SO_4, filtered and concentrated. The crude product was purified by silica column chromatography (hexanes/ethylacetate; 2:1) to give 740.0 mg of substance (8) in 64% yield. The product was used in consecutive steps without characterization. For synthesis of JMN5-165, a solution of substance (8) (supplement 1) (100.0 mg, 0.29 mmol) in CH_2Cl_2 (3.0 ml) was mixed with benzenesulfonyl chloride (40.0 mL, 0.31 mmol) and *i*-Pr_2NEt (80.0 mL, 0.46 mmol) and the reaction stirred for eight hours. $NaHCO_3$ (2.0 ml) was added to the reaction mixture, the organic layer separated and washed with brine (2.0 ml), then dried over Na_2SO_4 and concentrated. The crude product was purified by silica column chromatrography (hexanes/ethylacetate) to give a white solid that was dried under vacuum. ^{1}H NMR (400 MHz, $CDCl_3$, δ = 7.24 ppm); δ 9.56 (s, 1H), 8.17 (d, *J* = 8.0 Hz, 1H), 8.03-8.0 (m, 2H), 7.96-7.94 (m, 1H), 7.59-7.51 (m, 2H), 7.45-7.41 (m, 2H), 7.31-7.29 (m, 2H), 7.01-6.99 (m, 2H), 4.86 (q, *J* = 7.2 Hz, 1H), 2.23 (s, 3H), 1.68 (d, *J* = 7.6 Hz, 3H).

Synthesis of JMN5-166 followed the same protocol as described above for JMN5-165. ^{1}H NMR (400 MHz, $CDCl_3$, δ = 7.24 ppm); δ 10.02 (s, 1H), 8.26 (d, J = 8.4 Hz, 1H), 7.73-7.65 (m, 4H), 7.54-7.51 (m, 2H), 7.28-7.24 (m, 1H), 7.10-7.09 (broad, 1H), 7.06-7.02 (m, 2H), 6.70 (d, *J* = 8.4 Hz, 1H), 4.92 (q, *J* = 7.2 Hz, 1H), 2.25 (s, 3H), 1.65 (d, J = 7.2 Hz , 3H).
