# Supplementary figures and images for: Potent Host-Directed Small-Molecule Inhibitors of Myxovirus RNA-Dependent RNA-Polymerases
Source: PLoS One. 2011 May 16;6(5):e20069. doi: 10.1371/journal.pone.0020069 (PMC3095640; doi:10.1371/journal.pone.0020069)

**Figure S2**


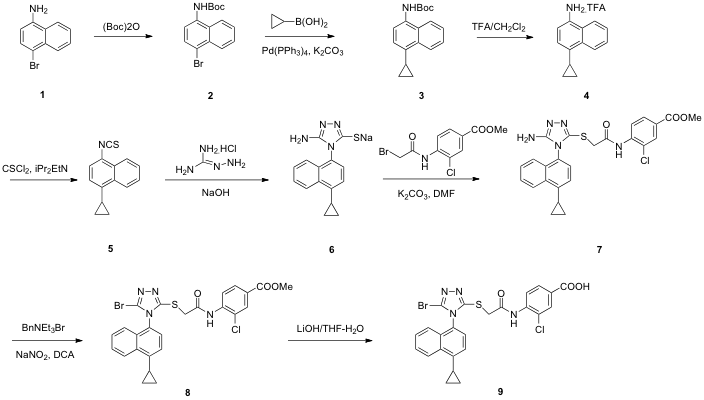

Supplement: Figure S2 — Structure of RDEA-806. (DOCX) [file pone.0020069.s002.docx]
